# A non-randomized comparative study of olfactory and gustatory functions in children who recovered from COVID-19 (1-year follow-up)

**DOI:** 10.3389/fped.2022.919061

**Published:** 2022-09-09

**Authors:** Leyla Namazova-Baranova, George Karkashadze, Irina Zelenkova, Elena Vishneva, Elena Kaytukova, Dina Rusinova, Natalia Ustinova, Natalia Sergienko, Yulia Nesterova, Leonid Yatsyk, Dmitrii Kratko, Svetlana Gubanova, Viktor Gankovskiy, Tina Gogberashvili, Tatiana Konstantinidi, Darya Bushueva, Anastasia Rykunova, Elena Shirdanina, Svetlana Sadilloeva, Natalia Sergeeva, Anastasia Lamasova, Elizaveta Leonova, Alina Pankova, Ekaterina Dubonosova

**Affiliations:** ^1^Pediatrics and Child Health Research Institute, Russian Scientific Center of Surgery Named After Academician B. V. Petrovsky, Moscow, Russia; ^2^Pirogov Russian National Research Medical University, Moscow, Russia; ^3^Department of Health, City Child Polyclinics No 133, Moscow, Russia

**Keywords:** COVID-19, children, olfactometry, cognitive functions, olfactory and gustatory functions

## Abstract

The experimental group included 68 children over 6 years of age who had recovered from COVID-19. The control group included 22 children over 6 years of age who have never had COVID-19. Research methods included neurological examination, verification of cognitive status, examination by an otolaryngologist, and smell and taste assessment. The examination was performed 6–8 weeks after COVID-19 recovery and after 1 year in some patients. Children who recovered from COVID-19 had a reduction in their ability to smell compared to children who had never had COVID-19. The olfactory thresholds and taste identification scores after recovery from COVID-19 were identical, whether the parents had reported anosmia in their children during COVID-19 or not, and irrespective of hyperthermia level and the presence or absence of headache and hyperhidrosis during COVID-19. Analysis of correlation with neuropsychiatric symptoms showed no differences in the olfactory thresholds in children irrespective of the presence of neuropsychiatric symptoms (tics, tremors, enuresis, compulsive movements, seizures, speech disorders, attention deficit, and easy fatigability) both in general, and in particular among subjects performing or not any compulsive movements, and experiencing or not a combination of easy fatigability and daytime sleepiness. Evidence suggests that in children and adolescents, partial hyposmia is associated with depressive symptoms, varying in severity from low to high, but symptoms of depression were not caused by COVID-19 infection itself. Analysis in subgroups with different degrees of state and trait anxiety did not reveal any significant differences in the olfactory threshold. A re-examination of 21 children was performed after 1 year. An objective olfactometric examination showed that the sensitivity to odorants increased significantly. In 1 year, we compared the thresholds of smell in children who had COVID-19 and those who did not have this disease: olfactory sensitivity after COVID-19 in children is restored to normal values. Schulte correction test showed that none of 14 children with asthenic manifestations in the form of fluctuations or exhaustion when performing the test immediately after COVID-19 had these manifestations after 1 year. Thus, asthenization of cognitive activity was recorded within the next 1.5 months after suffering from COVID-19 but was absent after 1 year.

## Introduction

Over the past few months, COVID-19 has been the focus of the global scientific community. As is well known, the olfactory disorder is considered a hallmark symptom of COVID-19 (1–4). Apparently, since children are less susceptible to SARS-CoV-2 infection, the main data currently available have been collected from cohorts of adult patients (5, 6). There is still no clarity about both the incidence (in the general population of patients with COVID-19, it can range from 32 to 69%) and the pathogenesis, as well as the outcomes of olfactory dysfunction in COVID-19 (6, 7). Research in pediatric populations is virtually non-existent worldwide; there is a paper on three pediatric COVID-19 cases where children developed anosmia and/or ageusia and an article about a multicenter study that included 10 children; yet there is no mention of any Russian studies (6, 8). We, therefore, planned and are currently conducting a study of the outcomes of olfactory disorders and associated gustatory disorders children develop during COVID-19 infection. This study is part of a larger study of the neurological outcomes of COVID-19.

When planning the study, we had a problem when selecting the method for assessment of the olfactory threshold. It is well known that the reaction to smell is mediated not only by the olfactory nerve, but also partially by the trigeminal nerve, and, possibly, by the glossopharyngeal nerve (9–11). In order to obtain information that might help to better understand the pathogenesis of olfactory dysfunction, we set the goal to investigate the olfactory threshold using three odorants, each of them being perceived *via* a different nerve. The olfactory tests most commonly used in studies worldwide are simple and convenient for clinical use, but as an assessment of the olfactory threshold, it is based on a single odorant, i.e., either n-butanol or phenyl ethyl alcohol (PEA) (12, 13). In this context, we decided to take advantage of the expertise of the Russian national school of thought in olfactometry. The method of olfactory threshold testing in adults patented by Russian authors in 1999 was taken as a basis for our research (14). The test kit required for this technique consists of serial dilutions of three odorants prepared under normal conditions, namely, (1) valerian root tincture (odor perception is mediated by the olfactory nerve), (2) acetic acid solution (odor perception is mediated by the olfactory and the trigeminal nerves), and (3) ammonia solution (odor perception is mediated by the olfactory, mainly the trigeminal, and possibly the glossopharyngeal nerve) (14).

Considering the extreme urgency of obtaining new information on the health consequences of the novel coronavirus infection, the number of subjects at the first stage of the study and, accordingly, the duration of the stage were reduced to the minimum acceptable values. The study is still underway. Due to the particular importance of the data, we present in this article the first interim results of our study.

### Purpose of the study

The purpose of the study was to assess smell and taste in children after recovery from COVID-19.

## Materials and methods

### Study design

The experimental group included children over 6 years of age who had recovered from COVID-19 and who showed no less than 2 weeks prior to the enrollment the first negative PCR test result for SARS-CoV-2 on nasopharyngeal or oropharyngeal swab samples, confirming the fact of recovery (in Russian clinical practice, two negative swab tests within 24 h are considered to be proof of recovery from COVID-19). The control group included children over 6 years of age who have never had COVID-19. Research methods included neurological examination, verification of cognitive status, examination by an otolaryngologist, and smell and taste assessment. To avoid excessive fatigue in children that might affect the results, the examination of each participant was carried out for 2 consecutive days. The examination was done immediately after COVID-19 recovery and after 1 year in some patients.

### Eligibility criteria

Inclusion criteria for the experimental group were as follows: age of 6–18 years; a confirmed COVID-19 case; 2–5 weeks from the date of the first negative PCR test result for SARS-CoV-2 on nasopharyngeal or oropharyngeal swab samples; and informed consent to processing of personal data signed by the parents or by the subjects aged 15 years and above.

Inclusion criteria for the control group were as follows: age of 6–18 years and informed consent to processing personal data signed by the parents or by the subjects aged 15 years and above.

Non-inclusion criteria for both groups were as follows: mental deficiency and severe neuropsychiatric disorders; acute inflammation of nasopharyngeal mucosa on the day of the study; acute respiratory infection; and exacerbation of allergic rhinitis with damage to the nasopharyngeal mucosa.

General exclusion criteria were as follows: acute respiratory infection; exacerbation of allergic rhinitis with damage to the nasopharyngeal mucosa; any persisting feeling of being unwell; and refusal to continue research due to technical difficulties, for example, new circumstances preventing the parents from accompanying their child to the research center.

### Study setting

The study was conducted at the Pediatrics and Child Health Research Institute, Central Clinical Hospital, Russian Academy of Sciences, Moscow. Examinations were performed in the first half of daylight hours. All subjects are Moscow residents.

### Primary outcome

The endpoints of the study are data on smell and taste sensitivity in the experimental group subjects. The primary outcomes of the study are (1) comparison of the average olfactory and gustatory sensitivity test scores in the experimental and control groups and (2) correlation between olfactory and gustatory sensitivity scores.

### Subgroup analysis

The experimental group was divided into two subgroups depending on the presence or absence of olfactory disorders during COVID-19, based on the results of parents’ questioning by the investigator. Similarly, the experimental group was divided into two subgroups according to the presence or absence of gustatory disorders during COVID-19 infection, as reported by the parents. Additional comparative analysis of target olfactory indicators between the subgroups was carried out.

The experimental group was divided into subgroups depending on the degree of fever, the presence or absence of headache, and the presence or absence of hyperhidrosis during COVID-19 infection. A comparative analysis of olfactory sensitivity characteristics between the subgroups was carried out.

The experimental group was divided into subgroups depending on the presence or absence of neuropsychiatric microorganic symptoms (tics, tremor, enuresis, compulsive movements, seizures, speech disorders, attention deficit, and easy fatigability). Similarly, the experimental group was divided into subgroups according to the presence or absence of easy fatigability and excessive daytime sleepiness and the presence or absence of compulsive movements. A comparative analysis of olfactory sensitivity characteristics between the subgroups was carried out.

The experimental group was divided into subgroups depending on different state and trait anxiety scores and depression rates. A comparative analysis of olfactory sensitivity characteristics between the subgroups was performed.

### Methods for identification of subjects meeting the non-inclusion and exclusion criteria

1. Clinical examination performed by a pediatrician, with the assessment of a child’s overall health status.
2. Clinical examination performed by a neurologist, with the assessment of the neuropsychiatric mental status. The findings were also used to record additional outcomes.
3. Assessment of cognitive abilities using the Wechsler Intelligence Scale for Children (WISC). The WISC is aimed at determining the individual IQ and consists of 12 subtests. Verbal and non-verbal intelligence is measured in points, and then the Full-Scale IQ is generated. The test was administered by a clinical psychologist.
4. Examination performed by the ENT physician to assess the oropharynx and nasopharynx in terms of the status of mucosa and patency of the upper respiratory tract.

### Outcome reporting methods

1. Interviewing the parents, one at a time, based on a specially developed checklist with 40 items to help identify the presence of clinical symptoms in a child with COVID-19. The results were entered into a special form. Parents were interviewed by a pediatrician.
2. Assessment of the sense of smell using the three-component olfactory test. This article presents the results of the assessment of one of the components, i.e., determination of the olfactory threshold. The olfactory threshold was tested as follows (15). The investigator presented the test tubes with serial dilutions of an odorant for 2–3 s, one at a time, to each nostril holding them at a distance of 2 cm from the subject’s nose. The subject was asked to take 1–2 short sniffs while drawing in the air through the nose more actively than during common breathing, and to tell the investigator whether he or she could smell anything. First, the odorant was presented at the lowest concentration (i.e., having the weakest odor). If the subject did not smell it, he or she was presented with the odorant at the next highest concentration (2 times higher), and then at the next highest concentration, the investigator gradually increased the odorant concentration until the subject was able to smell the odor. After the subject felt the smell, the subject was asked to confirm his or her choice by making the correct choice from three pairs of test tubes, one of them containing an odorant at a given concentration and the others containing a non-odorant substance. Distilled water was used as a non-odorant substance. The procedure of triple confirmation of the right choice was necessary to exclude false sensations, which can often occur in children. After the triple confirmation of the right choice in three pairs of test tubes, the detection threshold for every given odorant was considered to be identified. The lowest concentration level of an odorant the subject was able to detect is considered the olfactory threshold.
  The study began with establishing the olfactory threshold for dilutions of 20% alcohol tincture of valerian root, for a 70% aqueous solution of acetic acid, and then for a 10% aqueous solution of ammonia. The following dilutions of these odorants in distilled water (15 for each) were proposed: for 20% alcohol tincture of valerian root and 70% aqueous solution of acetic acid: 0.00015625, 0.0003125, 0.000625, 0.00125, 0.0025, 0.005, 0.01, 0.02, 0.04, 0.08, 0.16, 0.32, 0.64, 1.28, and 2.56%; for 10% aqueous ammonia solution: 0.000125, 0.00025, 0.0005, 0.001, 0.002, 0.004, 0.008, 0.016, 0.032, 0.064, 0.128, 0.256, 0.512, 1.024, and 2.048%. The following conditions of the study were observed: the study was carried out in a well-ventilated room with an exhaust duct, with no aggressive odors. The subject was asked to avoid any food for 60 min prior to the test. Neither the subject nor the investigator was allowed to wear perfumes. There were 60-s breaks between tests for different odorants. After determining the olfactory detection threshold for each odorant, the investigator entered the data into the protocol. The olfactory threshold was assessed in points, and each dilution level was assigned a number of points, namely, a minimum of 1 point for the highest concentration and a maximum of 12 points for the lowest one. The higher the score, the higher the subject’s ability to detect the odorant.

1. Assessment of taste sensitivity. The subject was asked to identify the taste of six different drinks. The composition of the drinks was selected based on the following requirements: ensure a maximum variety of taste sensations (acidity, sweetness, salinity, and bitterness) and recognizability of tastes for preschool and elementary school children, based on ethical considerations with regard to the young age of the subjects (preferably pleasant or neutral taste). The following drinks were presented in the order indicated: natural apple juice, water, chocolate cocktail, natural banana juice, milk, and natural cherry juice. The subjects were blindfolded and, therefore, unable to see the color of the liquid. Mechanical compression of the nasal passages was also done to exclude olfaction from participating in taste recognition. After that, the subject took a glass of liquid in his or her hands and tasted it, in no more than three sips. After identification of each liquid, the mouth was rinsed with water. Taste identification was a two-stage process. At stage I, the subject was asked to tell the taste of the liquid without any prompting. If there were difficulties or the answer was incorrect, stage II was proposed, i.e., forced choice of taste from the four proposed. The assessment was done as follows: 2 points for correct taste identification at stage I, 1 point for correct identification at stage II, and 0 points for incorrect taste identification. Taste identification data were entered into a special protocol, in which the points were summed up. The minimum possible total score was 0 and the maximum was 12. The composite score was the indicator of the child’s gustatory ability. The following test conditions were ensured: the test was carried out in a room with no strong odors. The subject was asked to avoid food for 60 min prior to the test. Before testing, the subject was to rinse his or her mouth with water. Neither the subject nor the investigator was allowed to wear any perfume. The study was carried out in the presence of one of the parents or another legal representative of the child, who was to make sure that the presented drinks were manufactured beverages and opened and poured into disposable glasses directly at the time of testing.
2. The Spielberger State-Trait Anxiety Inventory for Children (STAIC) adapted by Yu L. Khanin was used to detect the level of state and trait anxiety. The Spielberger-Khanin scale is designed for children aged 12 years and above. Rating points are scored, based on a 4-point Likert-type scale, with 4 composite score ranges corresponding to no anxiety, low anxiety, moderate anxiety, and high anxiety, respectively. The inventory was administered by a clinical psychologist.
3. The Beck Depression Inventory (BDI) is a depression rating scale designed to diagnose the level of depression. The subject responds to statements that are ranked with a gradual increase in the specific weight of a symptom in the overall severity of depression. Next, a composite score is calculated and the level of depression is determined (no depression, mild depression, moderate depression, and severe depression). The inventory was administered by a clinical psychologist.
4. The cognitive testing (Schulte correction test) was performed by a psychologist.

### Ethical review

Enrollment in the study was carried out upon receipt of a signed informed voluntary consent for examination from a parent or a legally authorized representative of the child, or by the child aged 15 years and above.

### Statistical analysis

Statistical analysis of the results was carried out using the STATISTICA version 11.0 software package (StatSoft Inc., Tulsa, United States). For the comparative assessment of the olfactory threshold values in the main analysis and in additional subgroups, the parametric Student’s *t*-test was used for independent samples. The significance level assumed was a *p*-value of < 0.05. To determine the relationship between the smell and taste characteristics, Pearson’s coefficient of linear correlation was used.

## Results

### Study subjects

A total of 68 children and adolescents, aged 6–18 years, were suggested for recruitment in the experimental group and 22 children of the same age were suggested for inclusion in the control group. After preliminary examinations, with the identification of subjects meeting the non-inclusion criteria, 64 participants were admitted to the experimental group and 20 participants were admitted to the control group. In the course of the study, 3 subjects were excluded from the experimental group due to the parents’ inability to take the child to the research site. The study was completed by 61 subjects in the experimental group and 20 subjects in the control group (Tables 1, 2).

**TABLE 1:** Characteristics of subjects in the two groups who completed the study.

	Experimental group	Control group
Number of subjects, *n*	61	20
Average age, years	11.4 ± 3.5	11.5 ± 3.7
Gender: female, %	38.7	45

**TABLE 2:** Additional characteristics of the experimental group subjects.

	Total	Males	Females
Time from laboratory-confirmed onset of COVID-19 to examination of study subjects, days	45.4 ± 4.6	–	–
Time from laboratory-confirmed recovery from COVID-19 (first negative swab test) to examination of study subjects, days	27.3 ± 3.2	–	–
Hyperthermia	81.5%	59.1%	40.9%
Asthenia during COVID-19	60.4%	69.7%	31.3%
Headache during COVID-19	53.7%	51.7%	48.3%
Anosmia or hyposmia during COVID-19	41.5%	63.6%	36.4%
Ageusia or hypogeusia during COVID-19	30.2%	56.2%	43.8%
Asymptomatic COVID-19	0%	–	–

### Main results

Note: the higher the score, the higher the olfactory sensitivity, and the lower the olfactory threshold (i.e., lower concentrations of the odorant can be detected).

The olfactory thresholds of all three odorants were significantly elevated in children with COVID-19 (Table 3). Thus, children who recovered from COVID-19 had a reduction in their ability to smell compared to children who had never had COVID-19. At the same time, the olfactory threshold for valerian root tincture was somewhat more elevated than the thresholds for the other two odorants (Table 3).

**TABLE 3:** The mean scores of olfactory threshold.

	Experimental group, points (min = 1, max = 12)	Control group, points (min = 1, max = 12)	*P*
Valerian root tincture	7.52 ± 1.68	9.05 ± 1.18	0.0008
Acetic acid solution	7.59 ± 1.79	8.79 ± 1.51	0.001
Ammonia solution	7.95 ± 2.27	9.58 ± 2.43	0.009

The higher the score, the higher the olfactory sensitivity, and the lower the olfactory threshold (i.e., lower concentrations of the odorant can be detected).

The ability to identify tastes is lower in children who recovered from COVID-19 (Table 4). Pearson’s coefficient of linear correlation between the olfactory thresholds and taste identification was *r* = 0.38, which means there was a weak positive correlation between the smell and taste characteristics. Significant gender differences were not fixed either by a sense of smell or taste.

**TABLE 4:** The mean scores of taste identification.

	Experimental group, points (min = 0, max = 12)	Control group, points (min = 0, max = 12)	*P*
Taste identification	8.05 ± 2.19	9.93 ± 1.83	0.003

### Additional results

The olfactory thresholds after recovery from COVID-19 were identical, whether the parents had reported anosmia in their children during COVID-19 or not (Table 5).

**TABLE 5:** Odor detection thresholds after recovery from COVID-19.

	Valerian root tincture, points	Acetic acid solution, points	Ammonia solution, points
Subjects who experienced olfactory disorders during COVID-19, as reported by parents	7.45 ± 1.37	7.68 ± 1.7	8.06 ± 1.99
Subjects with no reported olfactory disorders during COVID-19	7.54 ± 1.84	7.38 ± 2.08	7.81 ± 2.32

In addition, there were no differences in taste identification scores between these subgroups. However, even more remarkably, taste identification scores after recovery were similar in subgroups of subjects who did and did not experience the loss of taste during COVID-19 infection.

Based on the results of the analysis in additional subgroups, there was no difference in the olfactory threshold and taste identification after recovery, irrespective of hyperthermia level and of the presence or absence of headache and hyperhidrosis during COVID-19. Analysis of correlation with neuropsychiatric symptoms showed no differences in the olfactory thresholds and taste identification in children, irrespective of the presence of neuropsychiatric symptoms (tics, tremors, enuresis, compulsive movements, seizures, speech disorders, attention deficit, and easy fatigability) both in general, and in particular among subjects performing or not any compulsive movements, and experiencing or not a combination of easy fatigability and daytime sleepiness.

Analysis in subgroups with different levels of depression revealed certain significant differences (Table 6).

**TABLE 6:** Olfactory thresholds depending on the presence or absence of depression after recovery from COVID-19.

Assessment of depression using the Beck Depression Inventory	Valerian root tincture, points	Acetic acid aqueous solution, points	Ammonia water solution, points
Subjects with no depression	7.73 ± 1.46	8.32 ± 1.04	8.32 ± 1.68
Subjects with depression	6.29 ± 1.25	7.0 ± 2.64	8.14 ± 2.21
*P*	0.029	0.06	

Evidence suggests that in children and adolescents, partial hyposmia (lower olfactory sensitivity to the valerian root tincture odor and, to a lesser extent, acetic acid odor) is associated with depressive symptoms, varying in severity from low to high. At the same time, symptoms of depression are not caused by COVID-19 infection.

Analysis in subgroups with different degrees of state and trait anxiety did not reveal any significant differences in the olfactory threshold, although there was a tendency for a lower olfactory sensitivity to valerian tincture odor in the subgroup with high trait anxiety, compared to the subgroup with low trait anxiety (6.91 ± 1.3 vs. 7.95 ± 1.76 at *p* = 0.09).

We proposed a re-examination of 21 children after 1 year. None of the 9 children who had complaints of olfactory impairment at the initial examination had them at the second examination after 1 year. An objective olfactometric examination showed that the sensitivity to the three odorants increased significantly (from 5.6 to 10.4 points for valerian tincture, from 6.1 to 10.1 points for acetic acid, and from 6.6 to 9.8 points for ammonia alcohol). For 1 year, we compared the thresholds of smell in children who had COVID-19 and those who did not have this disease: among children who recovered more than 6 months ago, the thresholds of smell did not differ from those who did not get sick. Thus, olfactory sensitivity 6–12 months after suffering from COVID-19 in children was restored to normal values.

Cognitive testing (Schulte correction test) showed that none of the 10 children with asthenic manifestations in the form of fluctuations when performing the test immediately after COVID-19 had these manifestations after 1 year.

Thus, asthenization of cognitive activity, fixed by objective diagnostic methods, was recorded within the next 1.5 months after suffering from COVID-19 but was absent after 1 year. This indicates the absence of long-term consequences of COVID-19 on the cognitive activity of children in the surveyed sample but does not exclude emotional, personal, and social consequences that may follow unproductive educational activities during the period of asthenization of cognitive activity.

## Discussion

This study focused mainly on the assessment of the sense of smell but also taste in children with a history of COVID-19 infection. This is the first such study in Russia and one of the first in the world. Internationally, our study echoes a multicenter study (China, France, and Germany) with a slightly smaller sample size of 27 children (the main sample in that study was 367 adult participants) (6). Despite the differences in design, this multicenter study was the only one of interest for comparison of results by the time the manuscript was prepared. After a while, the results of the later studies appeared (16–19).

As reported by parents, 41.5% of children and adolescents suffering from COVID-19 had smell impairment and 30.2% had taste impairment. This is the very first data on the incidence of these disorders in Russian children, which indicates that despite the known milder course of COVID-19 in children, the olfactory and gustatory systems are widely affected by SARS-CoV-2. In the abovementioned international multicenter study, the incidence of both system disorders in children was more or less similar to our data and amounted to 37% (10 out of 27 children aged 6–17 years) (6).

The main result of our study is that based on objective examination approximately 4 weeks after recovery from COVID-19, children and adolescents had smell and taste disorders. Obviously, these changes persist after recovery from coronavirus. It is, therefore, important to assess the time to recovery of olfactory function and whether it is fully restored after recovery from COVID-19.

Another finding is that smell and taste after recovery from COVID-19 were equally affected in those who complained of impaired smell and taste during COVID-19 infection and those who did not. This indicates that complaints made by children and observations reported by parents do not fully reflect the smell dysfunction the former had. In the abovementioned international multicenter study, the authors also emphasize that 10 out of 90 participants (including one child) who underwent objective olfactory testing had olfactory dysfunction without any subjective olfactory complaints. There seems to be a cohort with extensive subclinical smell impairment, meaning that smell disorders in COVID-19 are more common than reported by patients.

No connection has been established between olfactory dysfunction and the presence of certain neurological symptoms. In addition, no connection was found between the olfactory function status and the severity of other symptoms of COVID-19 (hyperthermia, asthenia, headache, and hyperhidrosis). Colleagues from the international multicenter study came to the same conclusions, discovering that in different cohorts, correlations between olfactory dysfunction and the severity of COVID-19 were very different (6). This suggests that neither the severity of the disease nor the premorbid damage to the nervous system, but some different, unexplored factors, such as a specific viral strain, ethnicity, or phenotype, contribute to the onset of olfactory dysfunction in patients with COVID-19. In this regard, it is significant that in our study, children and adolescents with non-COVID-mediated depression experienced hyposmia. The relationship between depression and an altered sense of smell has been repeatedly shown in studies (20–22). In this case, it is important that this connection has been shown in a cohort of patients who recovered from COVID-19. Therefore, accordingly, as illustrated by depression, we can talk about the contribution of individual phenotypic factors to a person’s predisposition to olfactory disorders during COVID-19.

There is a weak positive relationship between the smell and taste status. This means a partial, but not a complete overlap of olfactory and gustatory dysfunction, which is confirmed by some clinical observations. It is possible that a single etiopathogenetic factor (infection with the SARS-CoV-2 virus) and the regional proximity of receptor localization determine the relationship between olfactory and gustatory disorders; at the same time, we see individual differences in susceptibility of the two systems to such pathology, which prevents a complete synchrony of both smell and taste disorders, though given the limitations of the study, this conclusion requires further verification.

## Limitations

A significant factor limiting the interpretation of the study results is the relatively small sample size. Even though the differences in study results are statistically significant, a larger sample size could increase the statistical significance of these findings.

Another limitation is retrospective questioning of parents and children about the symptoms of COVID-19 infection. On average, the survey was conducted 45 days after the onset of infection. This raises the question of the accuracy of data on the course of COVID-19 infection in children. When examining the sense of smell, we evaluated the olfactory threshold, which to a greater extent reflects the precognitive (or extra-cognitive) part of perception, while taste testing evaluated the taste identification ability, which largely reflects the cognitive part of perception. This is why it is somewhat improper to look for a direct correlation between taste and smell testing, and it must be taken into account when interpreting the results.

## Conclusion

In this study, we presented the first objective diagnostic data, indicating that in children and adolescents, changes in smell and taste sensitivity persist even 2–5 weeks after recovery from COVID-19. More importantly, these changes occur more often than reported by children and their parents. The involvement of olfactory sensitivity in the pathological process does not depend on the severity of the COVID-19 case or any premorbid neurological impairment. The study is still underway.

## Data availability statement

The raw data supporting the conclusions of this article will be made available by the authors, without undue reservation.

## Ethics statement

The studies involving human participants were reviewed and approved by Ethics Committee of CCH RAS. Written informed consent to participate in this study was provided by the participants’ legal guardian/next of kin.

## Author contributions

LN-B: idea and general heading. GK: idea, study design, and head of science. IZ: olfactory test – idea and examination. EV: literature search and database. EK, DR, and NU: patients management. NS, YN, LY, DK, SG, VG, TG, TK, DB, AR, ES, SS, NS, AL, EL, AP, and ED: patients examination, database filling, and statistics. All authors contributed to the article and approved the submitted version.
